# Effectiveness of home-based cardiac telerehabilitation programs in improving clinical outcomes after PCI: A systematic review and meta-analysis of randomized and non-randomized studies

**DOI:** 10.1016/j.ijcrp.2026.200679

**Published:** 2026-07-07

**Authors:** Sanjeet Kumar, Fnu Venjhraj, Ahmed W. Hageen, Javeria Farooq, Meva Ram, Fnu Sahil, Sandeep Kumar, Jugdesh Kumar, Muhammad Umar, Ravi Das, Sahil Jairamani, Mirza Mohammad Ali Baig, Carol Wright Becker

**Affiliations:** a Shaheed Mohtarma Benazir Bhutto Medical College, Lyari, Karachi, Pakistan; b Faculty of Medicine, Tanta University, Tanta, Egypt; c Liaquat University of Medical & Health Sciences, Jamshoro, Pakistan; d Sindh Institute of Child Health and Neonatology (SICHN), Sukkur, Pakistan; e Khairpur Medical College, Khairpur Mir's, Pakistan; f Jinnah Sindh Medical University, Karachi, Pakistan; g Islamic International Medical College, Riphah International University, Rawalpindi, Pakistan; h Department of Emergency Medicine, West Virginia University, Morgantown, WV, USA

**Keywords:** Home-based cardiac telerehabilitation, Cardiac rehabilitation, Telemedicine, Mobile health, mHealth, Percutaneous coronary intervention, Coronary artery disease

## Abstract

**Background:**

Home-Based Cardiac Telerehabilitation (HBCTR) is a potential alternative to center-based programs, but its effectiveness remains unclear. This meta-analysis evaluated the impact of this treatment on clinical outcomes and patient-reported quality of life (QoL).

**Methods:**

We searched Cochrane Central, Google Scholar, and PubMed until September 1, 2025, for studies evaluating HBCTR versus usual care. The primary clinical outcomes were 6 min walk test (6 MWT), VO_2_max, and left ventricular ejection fraction (LVEF). Outcomes were pooled as mean differences (MD), standardized mean differences (SMD) for QoL, and risk ratios (RR) for binary data using RevMan 5.4.1 (random-effects). Quality assessment was performed using the ROB-2 scale, and publication bias was analyzed using funnel plots.

**Results:**

Twelve studies comprising 1392 patients were included. HBCTR significantly improved 6MWT distance (MD: 18.76; 95% CI: 10.35–27.18; p < 0.0001), VO_2_max (MD: 3.53; 95% CI: 2.42–4.65; p < 0.00001), and LVEF (MD: 2.03; 95% CI: 0.15–3.91; p = 0.03). Significant reductions were observed in systolic blood pressure (MD: −3.09 mmHg; 95% CI: −5.50 to −0.69; p = 0.01), alongside improved medication adherence (RR: 1.25; 95% CI: 1.08–1.43; p = 0.002). QoL outcomes showed small and inconsistent improvements, while lipid parameters, anxiety, and depression demonstrated no consistent benefit.

**Conclusion:**

Compared to usual care, HBCTR improved functional capacity, systolic BP, VO_2_ max, LVEF, and adherence, while mental health and lipid outcomes remained inconclusive.

## Highlights

• HBCTR significantly improves functional capacity after PCI.
• VO_2_max and LVEF improved with home-based telerehabilitation.
• HBCTR reduced systolic blood pressure and improved medication adherence.
• Quality of life and lipid outcomes showed inconsistent benefits.
• HBCTR offers an effective alternative to center-based cardiac rehabilitation.

## 1 Introduction

Cardiovascular diseases remain the leading cause of morbidity and mortality worldwide, with patients surviving myocardial infarction (MI) or undergoing percutaneous coronary intervention (PCI) representing a high-risk population for recurrent events and rehospitalization. Acute myocardial infarction (AMI) is a severe type of coronary heart disease (CHD) and one of the leading causes of death and physical disability, particularly in the rapidly growing population of elderly persons [1]. Although PCI reduces mortality, enabling discharged patients to restore their health, returning to society remains a public health problem to be solved in the current situation [2]. Exercise-based cardiac rehabilitation (EBCR) is a cornerstone of post-PCI management because it improves exercise capacity, cardiac endurance, muscle strength, physical activity levels, and quality of life through organized exercise training, health education, and lifestyle modifications [3]. Moreover, EBCR promotes self-sufficiency and active living, lowering the risks of future cardiac events and mortality [3].

Over the last couple of years, there has been an advancement in the use of telemedicine and mobile health (mHealth) technologies in the field of cardiovascular medicine to enhance the delivery of new strategies for the prevention of cardiovascular risk factors. Digital health interventions, such as smartphone applications, wearable devices, and remote monitoring systems, provide continuous patient engagement, motivational intervention, daily health updates, and improved adherence to lifestyle modification and medical therapy [4]. These technologies have a high impact on physical activity, cardiovascular risk factor control, and long-term patient management in individuals with cardiovascular disease. A recent meta-analysis of digital health interventions following acute coronary syndrome (ACS) reported significant improvements in medication adherence, lifestyle modification, and secondary prevention outcomes, furthering the growing role of mHealth strategies in post-ACS and post-PCI care [5]. Moreover, studies from the LIGHT randomized clinical trial showed that the use of smart devices and mobile applications may help improve peak oxygen consumption (peak VO_2_), physical activity, and cardiovascular risk profiles in patients with greater cardiovascular risks [6]. These findings contribute to the expanding evidence base for digital health technologies to enhance long-term cardiovascular prevention and patient-centered care.

Advancements in telemedicine have unlocked a new way of delivering healthcare systems, home-based cardiac rehabilitation (HBCR) programs, as an accessible alternative to traditional center-based rehabilitation programs. Telemedicine-based cardiac rehabilitation uses different approaches, such as Internet-based platforms, wearable sensors, smartphones, and telemonitoring systems, to remotely deliver individualized strategies, such as exercise prescriptions, vital monitoring, ensuring medicine adherence, providing motivation, and continuous rehabilitation support [7]. Such approaches not only reduce logistical and financial barriers but also provide a self-controlled and easily operable approach that frequently limits participation in conventional cardiac rehabilitation programs. This adoption accelerated during the COVID-19 pandemic, which further increased the demand for remote and home-based healthcare delivery models, such as telemedicine-based cardiac rehabilitation [8]. Many previous systematic reviews and meta-analyses support the idea of telehealth-based cardiac rehabilitation, exploring its impact on exercise adherence, physical activity levels, and cardiovascular risk profiles, with outcomes comparable to those achieved in conventional rehabilitation programs [9]. However, despite the increasing evidence for the efficacy of telemedicine-based exercise rehabilitation, the efficacy of structured and individualized telerehabilitation interventions after MI or PCI is still not fully understood.

Therefore, this systematic review and meta-analysis synthesizes evidence from randomized and non-randomized controlled trials evaluating telerehabilitation-based interventions for secondary prevention after MI or PCI. The primary objective of this review was to determine whether these digital rehabilitation strategies reduce emergency cardiac care utilization and improve overall quality of life (QoL). Additionally, this study explored heterogeneity among trials according to patient adherence, telerehabilitation intervention type, and follow-up duration.

## 2 Method

A comprehensive review and meta-analysis were conducted to ascertain the effects of home-based cardiac telerehabilitation programs on post-PCI clinical outcomes. The protocol (PROSPERO ID: CRD420251141983) was registered prior to data extraction, and the meta-analysis and systematic review were conducted in accordance with the Preferred Reporting Items for Systematic Reviews and Meta-Analyses (PRISMA) Guidelines 2021 [10].

## 3 Literature search and search strategy

The research team used three databases, including PubMed, Google Scholar, and the Cochrane Library, to conduct a thorough search for published material. A structured search strategy combining controlled vocabulary (MeSH terms) and free-text terms related to ‘percutaneous coronary intervention’ and ‘home-based cardiac telerehabilitation’ was used, such as “PCI” OR “coronary angioplasty” and “home-based cardiac rehabilitation” OR “cardiac rehabilitation” OR “HBCTR”, “and we looked for studies published between the beginning of the study and September 1,2025. The complete database-specific reproducible search strategies are provided in Supplementary Appendix 1.1**.** A PRISMA 2021 flowchart was also documented for the study selection process.

### 3.1 Eligibility criteria

The Populations, Interventions, Comparisons, Outcomes, and Study Designs (PICOS) framework was used to describe the eligibility criteria. The inclusion criteria were as follows: 1) P – Adults with CHD, specifically patients after PCI, eligible for phase II cardiac rehabilitation; 2) I – Home-based cardiac telerehabilitation (HBCTR) delivered via mobile phones, tablets, computers, WeChat, or wearable devices. Typically included exercise prescription, telemonitoring, and telecoaching; 3) C – for control groups, because the nature of control groups varied substantially across studies (e.g., usual care, outpatient rehabilitation, center-based CR), this variability was expected to contribute to heterogeneity in the pooled results. Further details are provided in the characteristics table-1. 4) O – Primary outcome: physical function test, most often measured by 6-min walk test (6MWT), Vo2max, left ventricular ejection fraction (LVEF) and Secondary outcomes: overall quality of life (QoL), components of Qol with physical component summary (PCS) QoL, and mental component summary (MCS) QoL, systolic blood pressure, diastolic blood pressure, change of total cholesterol, triglyceride, low-density lipoprotein (LDL) cholesterol, high-density lipoprotein (HDL) cholesterol, anxiety, depression and medical adherence. 5) S – randomized controlled trials (RCTs) and controlled clinical trials (CCT). The search was limited to studies published in English.

**Table 1:** Baseline Characteristics of included studies.

Author, Year, Country	Design	Total (n)	IG (n, Male %)	CG (n, Male %)	Age IG (Mean ± SD)	Age CG (Mean ± SD)	Intervention	Control	Duration	Outcomes
Widmer et al., 2017 (USA)	RCT	71	29 (78%)	29 (85%)	62.5 ± 10.7	63.6 ± 10.9	DHI + CR	Standard CR	3 months	BP (SBP and DBP), lipid profile, Vo2max, QoL (overall), Depression
Dorje et al., 2019 (China)	RCT	312	156 (70%)	156 (70%)	59.1 ± 9.4	61.9 ± 8.7	SMART-CR/SP + WeChat	Usual care	12 months	6MWT, BP (SBP), lipid profile, QoL (PCS and MCS), Medication adherence, Depression, Anxiety.
Lee et al., 2013 (Korea)	RCT	55	26 (85%)	29 (76%)	54.3 ± 8.9	57.3 ± 7.5	HBCTR with pedometer + counseling	Usual care	3 months	BP (SBP and DBP), Quality of life, exercise capacity
Fang, 2019 (China)	RCT	67	33 (63.6%)	34 (61.8%)	60.2 ± 9.3	61.4 ± 10.7	HBCTR via belt strap, Smart phone and web port	Usual care	1.5 months	6MWT, BP (systolic blood pressure, diastolic blood pressure); anxiety and depression (CDS score); risk factors (FTND score); quality of life (SF-36 (PCS), SF-36 (MCS).
Li, 2022 (a) (China)	RCT	80	40 (67.5%)	40 (62.5%)	55.4 ± 8.9	55.6 ± 8.3	HBCTR via WeChat group and exercise bracelet	Outpatient rehabilitation	6 months	6MWT, QoL (overall), Medication adherence.
Zheng et al., 2024 (China)	RCT	106	53 (60%)	53 (56.6%)	63.5 ± 9.5	63.0 ± 10.4	HBCTR via wearing an intelligent sports bracelet.	Outpatient care and telephone follow-up	3 months	6MWT, quality of life (SF-36 (PCS), SF-36 (MCS), Vo2max, LVEF, Anerobic threshold.
Cruz-Cobo et al., 2024 (Spain)	RCT	300	150 (68.7%)	150 (69.3%)	61.1 ± 8.7	63.9 ± 8.4	HBCTR via eMOTIVA app installed on their mobile phones or tablets.	Usual care	3 months	BP (SBP and DBP), lipid profile, Adherence to the Mediterranean diet, Exercise capacity was measured with the 6MWT, Cardiovascular risk factors.
Bernal-Jiménez et al., 2021 (Spain)	RCT	128	67 (79%)	61 (64%)	57.7 ± 8.2	61.5 ± 9.5	mHealth group with app (EVITE project) in their phones which monitor food consumption, blood pressure, smoking, and therapeutic adherence.	Standard care	9 months	BP (systolic and diastolic); anxiety and depression (CDS score); risk factors (FTND score); quality of life (SF-36 (PCS), SF-36 (MCS); diet, frequency of food intake, Physical activity performed, smoking, knowledge of a healthy lifestyle
Xiaojie Li et al., 2023 (China)	RCT	50	26 (46.2%)	24 (50%)	66.0 ± 3.8	66.6 ± 4.4	HBCTR via wearable smart device with 5G IOT CR intelligence platform and Healthy Life Cycle application (app)	Traditional in-hospital CR training.	3 months	Lipid profile, Vo2max, anxiety and depression.
Li et al., 2022(b) (China)	RCT	95	47 (74.4%)	48 (81.3%)	65.3 ± 8.7	67.7 ± 7.6	Home-based online supervised exercise program (HOSEP) and WeChat group	Conventional health education and home exercise program booklet	1.5 months	6 MWT, Lipid profile, BP (systolic and diastolic).
Dehghani et al., 2024 (Iran)	RCT	80	40 (47.5%)	40 (50%)	49.8 ± 7.9	51.5 ± 7.5	Home-based CR (telerehabilitation)	Traditional center-based CR	2 months	health-related quality-of-life (PCS and MCS) BP (systolic and diastolic), LVEF.
Lee et al., 2017 (Korea)	CCT	48	22 (68.2%)	26 (84.6%)	55.9 ± 6.2	56.1 ± 7.0	Smart phone with"Our Family Heart Health Guardian” app, Smart band, KakaoTalk messages	Center-based CR	3 months	quality of life (SF-36 (PCS), SF-36 (MCS); medication adherence, regular exercise, stress, calorie intake, lipids, cholestero,

G = Intervention group; CG = control group; DHI = Digital health interventions; CR = cardiac rehabilitation; LVEF = Left ventricular ejection fraction; QoL = quality of life; SMART-CR/SP = a system involved smartphone-based home cardiac rehabilitation and secondary prevention program; 6MWT = 6-min walking test; HBCTR home-based cardiac telerehabilitation; SF-36 (MCS) SF-36 Health Survey (mental component summary scale); SF-36 (PCS) SF-36 Health Survey (physical component summary scale); IOT = internet of things; RCT = Randomized controlled trial; CCT=Controlled clinical trial.

The exclusion criteria were as follows: 1) protocol study, 2) irrelevant outcomes, 3) crossover trial, and 4) different follow-up.

### 3.2 Study quality and risk of bias

**Fig. 1:**
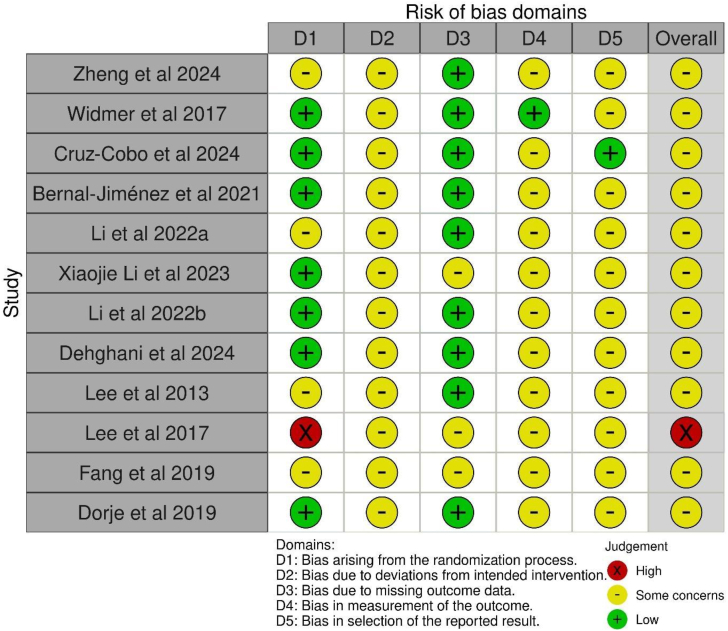
Risk of bias assessment of the included studies using the Cochrane Collaboration Risk of Bias 2 (RoB 2) tool.

SK and JF, two researchers, used the Cochrane risk of bias tool 2.0 to independently evaluate the methodological quality. The five bias domains— Randomization process (D1), Deviations from intended intervention (D2), Missing outcome data (D3), Measurement of the outcome (D4), Selection of the reported result (D5)—were used to evaluate the quality assessment of the study (11). It is the most widely used tool (approximately 100% for Cochrane reviews and 31% for non-Cochrane reviews), and its bias domains were chosen to intentionally cover all basic bias mechanisms in RCTs [11]. The study-specific judgments and rationale for each domain are presented in Fig. 1. Formal assessment of publication bias was not possible because no individual outcome included the minimum number of studies (n = 10) generally recommended for funnel plot interpretation.

### 3.3 Data extraction

All study data were extracted independently by two authors (MR and SK), and any disagreements were resolved by discussion with a third author (FV). Key information about the trial participants at baseline (sample size and age) and authors (name and year) was gathered. Results were also extracted, with the physical function test (6 MWT), Vo2max, and LVEF as the primary outcomes and overall QoL, PCS QoL, MCS QoL, systolic and diastolic blood pressure, change in total cholesterol, triglycerides, LDL cholesterol, HDL cholesterol, medical adherence, anxiety, and depression as secondary outcomes. We acknowledge that converting medians and IQRs to means and SDs using Pudar Hozo and Wan's methods [12,13] introduces estimation errors, and results involving transformed data should therefore be interpreted with caution.

### 3.4 Statistical analysis

Statistical analyses were conducted using Review Manager (V.5.4.1 Cochrane Collaboration, London, UK). Given the anticipated heterogeneity among the intervention and control groups, we conducted leave-one-out sensitivity analyses to assess the stability of the pooled estimates. For dichotomous outcomes we use risk ratio (RR) and for continuous outcomes we use mean difference (MD) were calculated for continuous outcomes with 95% confidence intervals (CIs). For QoL, we used the standardized mean difference (SMD) due to the different scales used in QoL (SF-12, SF-36, Dartmouth). All results were assessed using a random-effects model. Using Higgins I^2^ statistics, the heterogeneity across pooled studies was evaluated. A value of I^2^ = 25%-50% was regarded as mild heterogeneity, 50%-75% as moderate, and more than 75% as severe [14]. Instances where pooled effects were highly sensitive to individual studies were noted as potentially unstable. Since our meta-analysis included more than 10 studies, we generated funnel plots to assess publication bias. A p-value <0.05 was considered significant throughout the analysis.

### 3.5 Subgroup analyses

Subgroup analyses were performed to explore the sources of heterogeneity based on (1) intervention type (exercise-based HBCTR vs. digital-monitoring interventions) and (2) comparator type (passive usual care vs. active center-based/outpatient cardiac rehabilitation). Subgroup analyses were conducted for quality-of-life outcomes (PCS and MCS), systolic blood pressure, depression, and lipid profile outcomes, where sufficient studies were available. Given that only a single CCT was included, a formal subgroup analysis by study design was not feasible, as a subgroup requires a minimum of two studies to estimate within-group variance. Instead, we conducted a sensitivity analysis excluding this CCT and reran the pool using RCT-only data. In addition, subgroup analysis based on follow-up duration was not feasible because only one study (Bernal-Jiménez et al., 2021) had a follow-up duration of 9 months, which differed from the remaining studies. However, a sensitivity analysis was performed in which this study was analyzed separately to assess its impact on different outcomes. The findings were narratively described alongside pooled estimates to provide context regarding long-term outcomes. This study was not included in the pooled subgroup analysis or the test for subgroup differences in accordance with methodological recommendations discouraging single-study subgroups.

#### 3.5.1 Grade assessment

Two researchers (FV and SK) independently assessed the evidence quality using the GRADE profiler software and conducted a detailed assessment using a range of core dimensions, including study design, risk of bias, inconsistency, and indirectness. The quality of the evidence was classified into four levels based on the GRADE criteria: High Quality— there is a high level of confidence that the computed value is near the true value; Moderate Quality— there is some degree of confidence in the computed value, but the actual value may differ significantly from the computed value; Low Quality— there is little confidence in the calculated value; and Very Low Quality— there is a very low level of confidence in the measured variable. The grading of the quality of the evidence will be combined with the size of the effect for analysis, according to the Cochrane Handbook for meta-analysis.

## 4 Results

### 4.1 Study selection and characteristics

**Fig. 2:**
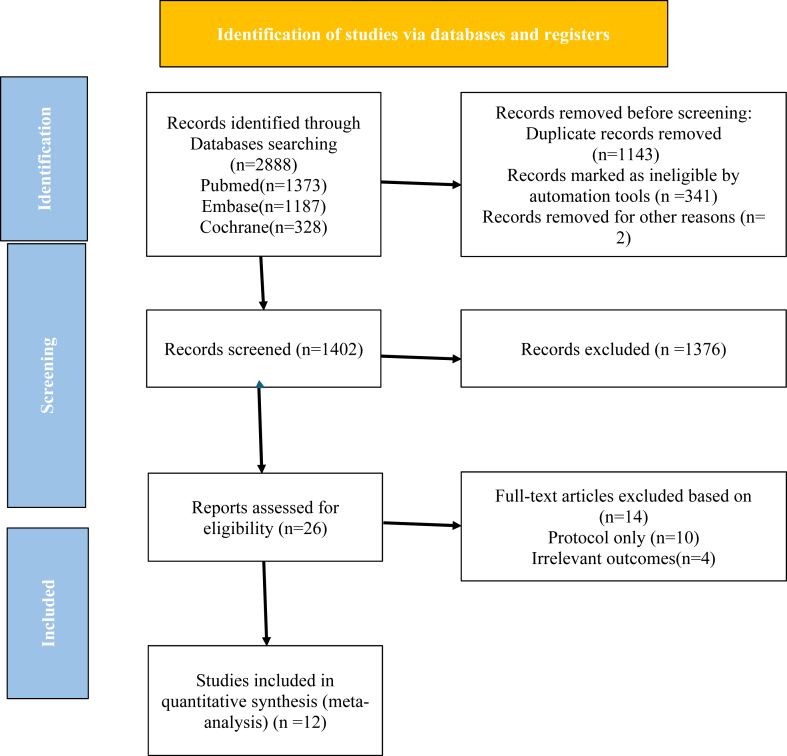
**PRISMA 2021 flow diagram.** Illustration of the study selection process. Out of 2888 identified records, 1404 were screened, 26 were assessed for full eligibility, and 12 studies (n = 1392 participants) were included in the final meta-analysis.

A comprehensive literature search was conducted and yielded 2888 articles (1373 from PubMed, 1187 from Google Scholar, and 328 from the Cochrane Library), of which 1143 duplicate studies and 341 ineligible studies and case report articles were removed. A total of 1376 studies were excluded for not meeting the inclusion criteria. Only 26 records were identified as eligible after the initial screening of the titles and abstract of 1402 documents from 26 full-text papers screened. At the same time, only 12 articles fulfilled the absolute eligibility criteria, and all 12 articles comprising 1392 patients (697 in HBCTR group and 695 in control group) could be included in the quantitative meta-analysis. A PRISMA 2021 flowchart documenting the study selection process and a corresponding PRISMA checklist are provided in Fig. 2 and Supplementary Table 1 summarizes all the included studies, types of HBCTR interventions, control, study duration and outcomes of all the studies.

### 4.2 Quality assessment

Across 12 studies, most domains demonstrated either low risk of bias or some concerns, with only one study (Lee et al., 2017) judged to have a high overall risk of bias. Regarding bias arising from the randomization process (D1), most studies showed low risk or some concerns, although Lee et al. (2017) presented a high risk due to problems with randomization. For bias due to deviations from the intended intervention (D2) and bias due to missing outcome data (D3), most studies raised some concerns but did not reach a high-risk judgment, suggesting that attrition and protocol deviations were generally manageable. In terms of bias in the measurement of the outcome (D4), most studies were rated as having some concerns, reflecting potential issues with blinding of outcome assessment. The majority of studies showed low risk for the bias of reported result (D5) and some have insufficiently detailed protocols, so they were also judged.

Overall, the risk of bias assessment showed that most of the studies were at low to moderate risk of bias, and some domains showed concerns that could be judged. One study showed a high risk of bias, which limited the pooled conclusions.

#### 4.2.1 GRADE assessment

The certainty of the evidence for different outcomes ranged from moderate to very low according to the GRADE evaluation, as summarized in Supplementary Table 2. Compared with standard care, HBCTR showed significant improvements in exercise capacity (6 MWT), quality of life (PCS and MCS), systolic blood pressure, LVEF, VO 2 max, and medication compliance. In contrast, the effects on lipid profiles, anxiety, depression, and diastolic blood pressure were minimal or not statistically significant. Most other results had low or very low certainty. Moderate-certainty evidence supported improvements in LVEF and systolic blood pressure. This was due to methodological limitations; lack of blinding, which is common in telerehabilitation studies; large heterogeneity; small sample sizes; and wide confidence intervals. In conclusion, HBCTR appears to be beneficial to improve functional and specific cardiovascular outcomes after cardiac events, but further high-quality randomized trials are needed to improve the certainty of the evidence.

### 4.3 Overall results

HBCTR consistently improved medical adherence and physical function, such as 6-min walk distance and VO_2_ max, across the 12 included trials. These results were corroborated by other investigations with generally strong conclusions. Improvements in systolic blood pressure and LVEF were statistically but not clinically significant. There was little trust in the results because the effects on QoL, diastolic blood pressure, lipid profiles, and psychological outcomes were typically minor, inconsistent, based on one or two trials, and frequently showed high heterogeneity. Given the substantial heterogeneity, caution is warranted in interpreting the pooled effects, and the appropriateness of combining these studies is limited. Sensitivity analyses (leave-one-out analysis) showed that several aggregated results were unstable and susceptible to individual studies. While the evidence for other cardiometabolic and psychosocial outcomes is still unclear and should be interpreted cautiously, HBCTR generally shows promise for enhancing functional capacity and adherence.

### 4.4 Primary outcomes

#### 4.4.1 Physical function test

**Fig. 3:**
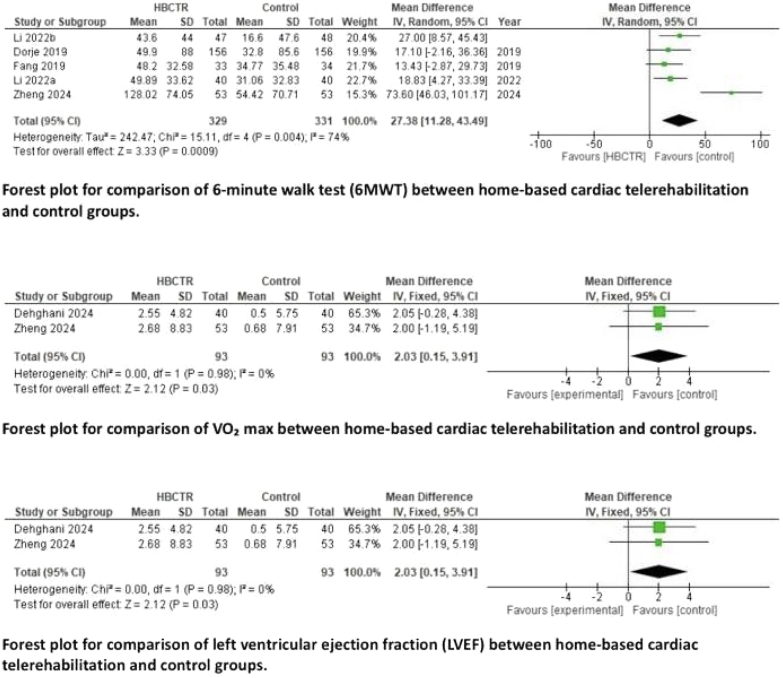
Primary outcomes; 6 MWT, LVEF and VO2 max.

Five studies analyzing the outcomes of the 6 MWT showed a significant improvement in the HBCTR group compared to the control group. (MD: 27.38, Cl: [11.28, 43.49], p = 0.0009, I^2^ = 74%). By excluding Zheng 2024, heterogeneity was reduced to 0%, indicating robust results (MD: 18.76, Cl: [10.35, 27.18], p=<0.0001, I^2^ = 0%). Given the substantial heterogeneity, caution is warranted in interpreting pooled effects, and the appropriateness of combining these studies is limited (Fig. 3).

#### 4.4.2 VO2 max

Three studies revealed significant improvements in the HBCTR group compared to the control group. (MD: 2.55, Cl: [0.42, 4.68], p = 0.02, I^2^ = 91%). Due to high heterogeneity, caution is warranted, and by performing a leave-one-out analysis (excluding Widmer 2017), it was reduced to 43% while maintaining a significant effect (MD: 3.53, Cl: [2.42, 4.65], p < 0.00001, I^2^ = 43%) (Fig. 3).

#### 4.4.3 Left ventricular ejection fraction (LVEF)

2 studies showed significant improvement in HBCTR group vs control (MD: 2.03, Cl: [0.15, 3.91], p = 0.03, I^2^ = 0%). This outcome is informed by only two studies; therefore, the findings should be interpreted cautiously due to limited evidence (Fig. 3).

### 4.5 Secondary outcomes

#### 4.5.1 Quality of life

This outcome was supported by only 2 studies, so the results should be interpreted cautiously due to limited evidence. Other studies measure components of QoL which include physical and mental component summary.

#### 4.5.2 Physical component summary of QoL

**Fig. 4:**
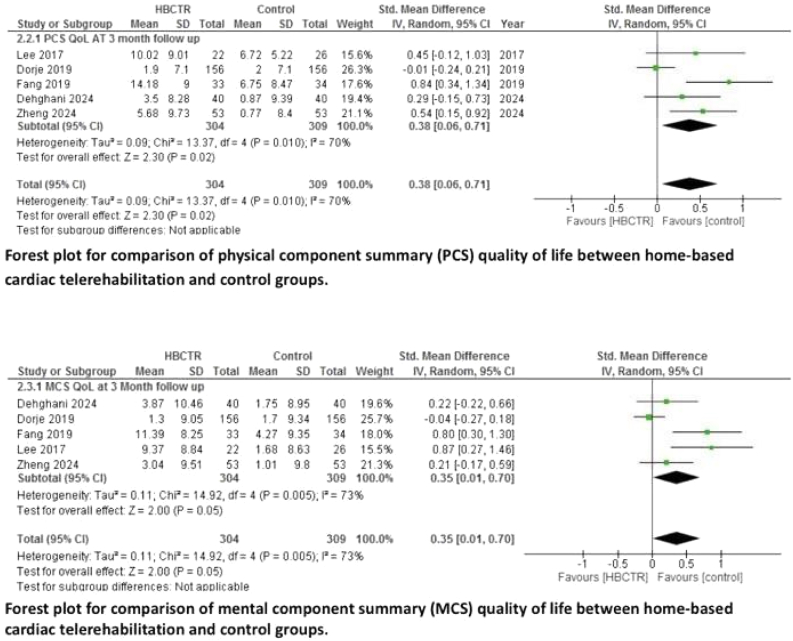
Quality of life; PCS and MCS.

For PCS, pooled analysis of 5 studies with 3-month follow-up demonstrated a small-to-moderate statistically significant improvement in the intervention group compared with controls (SMD = 0.38, 95% CI 0.06 to 0.71, p = 0.02; I^2^ = 70%). These findings indicate that the intervention was associated with improved physical aspects of QoL in the short term (Fig. 4).

Given that Dorje et al. (2019) used the SF-12 to assess QoL while the remaining studies employed the SF-36 instruments that share a Physical Component Summary score but differ in responsiveness to change a sensitivity analysis was performed excluding this study. This reduced within-subgroup heterogeneity from I^2^ = 70% to 0%, with the pooled SMD remaining statistically significant and directionally consistent with the main analysis. These findings suggest that measurement instrument heterogeneity is a contributing source of statistical variance in this outcome, and results should be interpreted accordingly.

Subgroup analysis by intervention type showed a significant difference for quality of life (PCS) at 3 months (p = 0.13). Exercise-based HBCTR (3 studies, N = 253) demonstrated a significant improvement (SMD = 0.54, 95% CI 0.25 to 0.83; I^2^ = 22%), while digital monitoring–only interventions (2 studies, N = 360) showed no significant effect (SMD = 0.14, 95% CI −0.29 to 0.57; I^2^ = 54%). Overall, HBCTR interventions improved PCS (SMD = 0.38, 95% CI 0.06 to 0.71; p = 0.02), with substantial heterogeneity (I^2^ = 70%). (Supplementary Fig. 1).

#### 4.5.3 Mental component summary of QoL

At 3-month follow-up, pooled analysis of 5 studies demonstrated a borderline statistically significant effect favoring the HBCTR group compared with controls (SMD = 0.35, 95% CI 0.01 to 0.70, p = 0.05; I^2^ = 73%) (Fig. 4). However, substantial heterogeneity was observed. Leave-one-out sensitivity analysis excluding Fang et al. (2019) reduced heterogeneity (I^2^ = 65%) and altered the effect estimate (SMD = 0.24, 95% CI –0.09 to 0.56, p = 0.15), suggesting that the findings should be interpreted cautiously.

Only one study reported mental component summary outcomes at 9 months (SMD = −0.32, 95% CI –0.67 to 0.03, p = 0.07). Therefore, the 9-month follow-up was not included in the quantitative pooled analysis due to limited evidence and the potential for biased estimation from a single study. Instead, the 9-month findings were interpreted narratively and suggested that the beneficial effects observed at shorter follow-up durations may diminish over time.

Subgroup analysis by intervention type showed no significant difference for quality of life (MCS) at 3 months (p = 0.98). Exercise-based HBCTR (3 studies, N = 253) showed a significant improvement (SMD = 0.38, 95% CI 0.03 to 0.74; I^2^ = 50%), while digital monitoring–only interventions (2 studies, N = 360) showed no significant effect (SMD = 0.37, 95% CI -0.52 to 1.26; I^2^ = 87%). Overall, HBCTR showed a borderline significant improvement in MCS (SMD = 0.35, 95% CI 0.01 to 0.70; p = 0.05), with substantial heterogeneity (I^2^ = 73%). (Supplementary Fig. 2).

#### 4.5.4 Systolic blood pressure

**Fig. 5:**
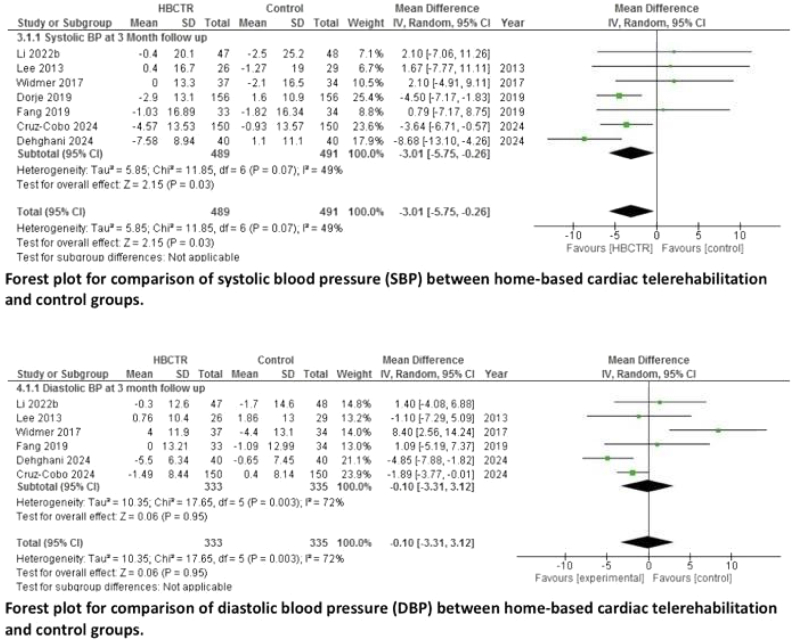
Blood pressure; SBP and DBP.

At 3-month follow-up, pooled analysis of 7 studies demonstrated a statistically significant reduction in systolic blood pressure favoring the HBCTR group compared with controls (MD = −3.01 mmHg, 95% CI –5.75 to −0.26, p = 0.03; I^2^ = 49%) (Fig. 5). However, although statistically significant, the magnitude of reduction was clinically modest. Only one study reported SBP outcomes at 9 months (MD = −2.64, 95% CI –8.60 to 3.32, p = 0.39). Therefore, the 9-month follow-up was not included in the quantitative pooled analysis due to limited evidence and the potential for biased estimation from a single study. Instead, the 9-month findings were interpreted narratively after conducting separate analyses including the 9-month follow-up, which suggested limited long-term benefit. Consequently, quantitative pooled analysis was restricted to studies reporting 3-month outcomes, where HBCTR demonstrated more consistent short-term improvement in systolic blood pressure.

Subgroup analysis by intervention type showed no significant subgroup differences for systolic blood pressure (p = 0.81). Exercise-based HBCTR (5 studies, N = 597) showed a pooled MD of −2.92 mmHg (95% CI −6.90 to 1.06; I^2^ = 55%), while digital monitoring interventions (2 studies, N = 383) showed a pooled MD of −2.03 mmHg (95% CI −8.29 to 4.23; I^2^ = 66%). Although subgroup estimates were non-significant, the overall pooled effect remained significant (MD = −3.01 mmHg, 95%CI -5 75 to −0.26, p = 0.03), i^2^ 49% Residual heterogeneity within the exercise-based subgroup may reflect variability in smaller studies. (Supplementary Fig. 3).

#### 4.5.5 Diastolic blood pressure

At 3-month follow-up, pooled analysis of 6 studies demonstrated no significant difference in diastolic blood pressure between the HBCTR and control groups (MD = −0.10 mmHg, 95% CI –3.31 to 3.12, p = 0.95; I^2^ = 72%), with substantial heterogeneity observed across studies. Leave-one-out sensitivity analysis identified Widmer et al. (2017) as the primary contributor to heterogeneity (Fig. 5). The divergent findings in this study may be explained by methodological differences, including the use of a high-intensity center-based cardiac rehabilitation comparator and a digital-health adjunct intervention rather than a standalone home-based programme. Exclusion of Widmer et al. reduced heterogeneity to I^2^ = 32% and changed the pooled estimate to MD = −1.95 mmHg (95% CI –4.03 to 0.12, p = 0.07), although the result remained statistically insignificant.

Only one study reported diastolic blood pressure outcomes at 9 months (MD = −2.90, 95% CI –6.75 to 0.95, p = 0.14). Therefore, the 9-month follow-up was not included in the quantitative pooled analysis because of limited evidence and the potential for biased estimation from a single study. Instead, the 9-month findings were interpreted narratively after conducting separate analyses including the 9-month follow-up, which demonstrated limited evidence of long-term benefit. Given the persistent heterogeneity and non-significant pooled estimates, no reliable conclusion regarding the effect of HBCTR on diastolic blood pressure can currently be drawn.

#### 4.5.6 Total cholesterol change

**Fig. 6:**
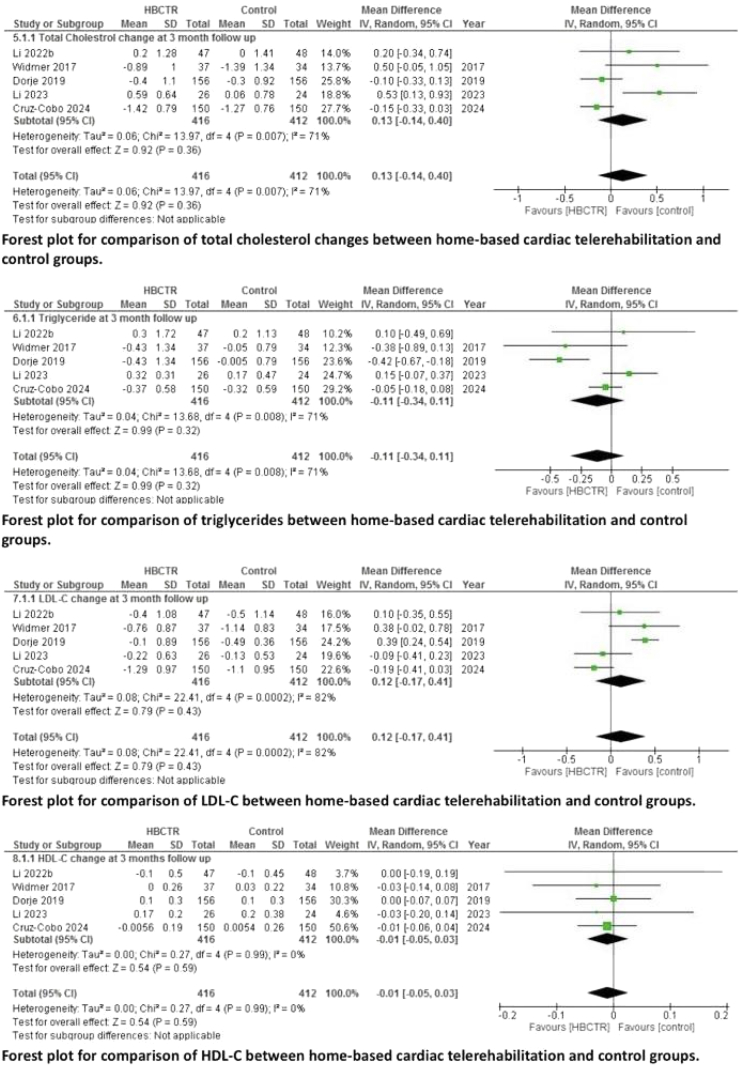
Lipid Profile; total cholesterol change, Triglycerides, LDL-C and HDL-C.

At 3-month follow-up, pooled analysis of 5 studies demonstrated no significant difference in total cholesterol between the HBCTR and control groups (MD = 0.13 mmol/L, 95% CI –0.14 to 0.40, p = 0.36; I^2^ = 71%), with substantial heterogeneity observed across studies (Fig. 6). The heterogeneity appeared to be driven by variation in effect direction between studies, as Li et al. (2023) and Widmer et al. (2017) reported modest increases in total cholesterol in the HBCTR group, whereas Dorje et al. (2019) and Cruz-Cobo et al. (2024) demonstrated neutral or marginally favorable effects. These differences may reflect variability in intervention components, particularly the inclusion of dietary counseling within HBCTR programmes.

Leave-one-out sensitivity analysis excluding Li et al. (2023) reduced heterogeneity to I^2^ = 49%, while the pooled effect remained statistically insignificant (MD = −0.01 mmol/L, 95% CI –0.23 to 0.21, p = 0.92). As the pooled estimates changed following exclusion of individual studies, the findings for this outcome appear unstable and should therefore be interpreted cautiously.

Only one study reported total cholesterol outcomes at 9 months (MD = −0.49 mmol/L, 95% CI –0.89 to −0.09, p = 0.02). Therefore, the 9-month follow-up was not included in the quantitative pooled analysis because of limited evidence and the potential for biased estimation from a single study. Instead, the 9-month findings were interpreted narratively after conducting separate analyses including the 9-month follow-up, suggesting possible longer-term improvement in total cholesterol with HBCTR; however, this observation requires confirmation in larger studies with longer follow-up durations.

Subgroup analysis by intervention type showed no significant differences for total cholesterol at 3 months (p = 0.31). Exercise-based HBCTR (3 studies, N = 445) showed no effect (MD = 0.17 mmol/L, 95% CI −0.30 to 0.63; I^2^ = 80%), while digital monitoring–only interventions (2 studies, N = 383) also showed no effect (MD = −0.10 mmol/L, 95% CI −0.32 to 0.13; I^2^ = 0%). Overall, there was no significant effect (MD = 0.06 mmol/L, 95% CI −0.20 to 0.32; p = 0.65), with moderate heterogeneity (I^2^ = 62%) (Supplementary Fig. 4).

#### 4.5.7 Triglyceride

At 3-month follow-up, pooled analysis of 5 studies demonstrated no significant difference between the HBCTR and control groups, although a slight improvement favoring HBCTR was observed (MD = −0.11, 95% CI –0.34 to 0.11, p = 0.32; I^2^ = 71%). Substantial heterogeneity was present across studies (Fig. 6). Leave-one-out sensitivity analysis excluding Dorje et al. (2019) substantially reduced heterogeneity (I^2^ = 34%) while producing a similar non-significant effect estimate (MD = −0.01, 95% CI –0.17 to 0.16, p = 0.94), indicating that the overall findings remained robust despite study-related heterogeneity.

Only one study reported outcomes at 9 months (MD = −0.01, 95% CI –0.34 to 0.32, p = 0.95). Therefore, the 9-month follow-up was not included in the quantitative pooled analysis because of limited evidence and the potential for biased estimation from a single study. Instead, the 9-month findings were interpreted narratively after conducting separate analyses including the 9-month follow-up, which demonstrated insufficient evidence to draw definite conclusions regarding long-term effects.

Subgroup analysis by intervention type showed a significant difference for triglycerides (TG) at 3 months (p = 0.001). Exercise-based HBCTR (3 studies, N = 445) showed no significant effect (MD = 0.02 mmol/L, 95% CI −0.12 to 0.16; I^2^ = 16%), while digital monitoring–only interventions (2 studies, N = 383) significantly reduced TG (MD = −0.42 mmol/L, 95% CI −0.64 to −0.20; I^2^ = 0%). Overall, there was no significant effect (MD = −0.11 mmol/L, 95% CI −0.34 to 0.11; p = 0.32), with substantial heterogeneity (I^2^ = 71%) (Supplementary Fig. 5).

#### 4.5.8 LDL-C change

At 3-month follow-up, pooled analysis of 5 studies demonstrated no significant difference in LDL-C levels between the HBCTR and control groups (MD = 0.12, 95% CI –0.17 to 0.41, p = 0.43; I^2^ = 82%), with substantial heterogeneity observed across studies (Fig. 6). Leave-one-out sensitivity analysis excluding Dorje et al. (2019) reduced heterogeneity to a moderate level (I^2^ = 55%) while the pooled effect remained statistically insignificant (MD = 0.01, 95% CI –0.24 to 0.26, p = 0.93), suggesting that the overall findings were not materially altered by exclusion of the study.

Only one study reported LDL-C outcomes at 9 months and demonstrated a significant reduction favoring HBCTR (MD = −0.59, 95% CI –0.93 to −0.25, p = 0.0007). However, because this finding was derived from a single study, the 9-month follow-up was not included in the quantitative pooled analysis due to limited evidence and the potential for biased estimation. Instead, the 9-month findings were interpreted narratively after conducting separate analyses including the 9-month follow-up, suggesting possible longer-term benefit of HBCTR on LDL-C levels; however, further studies with longer follow-up durations are required to confirm this observation.

Subgroup analysis by intervention type showed significant differences for LDL-C at 3 months (p < 0.00001). Exercise-based HBCTR (3 studies, N = 445) showed no significant effect (MD = −0.12 mmol/L, 95% CI −0.29 to 0.04; I^2^ = 0%). Digital monitoring–only interventions (2 studies, N = 383) favored usual care, with a significant MD of 0.39 mmol/L (95% CI 0.25 to 0.53; I^2^ = 0%). Overall, there was no significant effect (MD = 0.12 mmol/L, 95% CI −0.17 to 0.41; p = 0.43), with substantial heterogeneity (I^2^ = 82%) (Supplementary Fig. 6).

#### 4.5.9 HDL-C change

At 3-month follow-up, pooled analysis of 5 studies demonstrated no significant difference in HDL-C levels between the HBCTR and control groups (MD = −0.01, 95% CI –0.05 to 0.03, p = 0.59; I^2^ = 0%), with effects slightly favoring the control group (Fig. 6). Only one study reported HDL-C outcomes at 9 months (MD = −0.04, 95% CI –0.14 to 0.07, p = 0.52). Therefore, the 9-month follow-up was not included in the quantitative pooled analysis because of limited evidence and the potential for biased estimation from a single study. Instead, the 9-month findings were interpreted narratively after conducting separate analyses including the 9-month follow-up, which also demonstrated no significant long-term effect. Overall, the available evidence does not suggest a meaningful effect of HBCTR on HDL-C levels.

Subgroup analysis by intervention type showed no significant differences for HDL-C at 3 months (p = 0.92). Exercise-based HBCTR (3 studies, N = 445) showed no effect (MD = −0.01 mmol/L, 95% CI −0.06 to 0.04; I^2^ = 0%), while digital monitoring–only interventions (2 studies, N = 383) also showed no effect (MD = −0.01 mmol/L, 95% CI −0.07 to 0.05; I^2^ = 0%). Overall, there was no significant effect on HDL-C (MD = −0.01 mmol/L, 95% CI −0.05 to 0.03; p = 0.59), with no heterogeneity (I^2^ = 0%) (Supplementary Fig. 7).

#### 4.5.10 Medical adherence

**Fig. 7:**
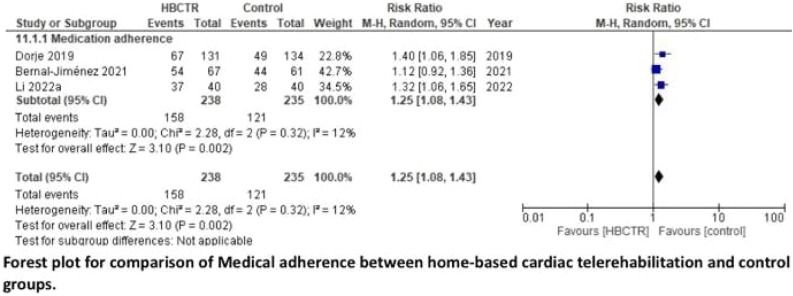
Medical adherence.

Three studies showed medical adherence to prescribed therapies and consistently demonstrated higher adherence in the HBCTR group compared with controls (RR: 1.25, 95% CI [1.08, 1.43], p = 0.002) with low heterogeneity (I^2^ = 12%), indicating a robust positive effect of HBCTR interventions on patient compliance (Fig. 7).

#### 4.5.11 Anxiety

**Fig. 8:**
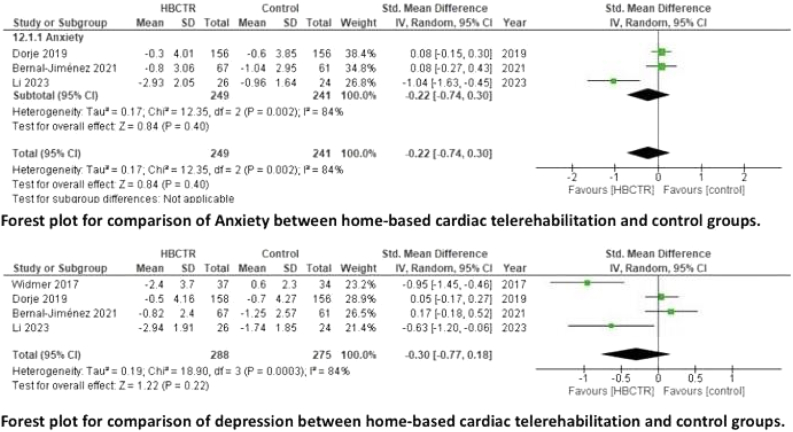
Anxiety and depression.

Anxiety outcomes were reported in 3 studies and showed no significant improvements with high heterogeneity (SMD: −0.22, Cl: [−0.74, 0.30], p = 0.40, I^2^ = 84%). Excluding Li 2023 reduced the heterogeneity with insignificant results (SMD: 0.08, Cl: [−0.11, 0.26], p = 0.42, I^2^ = 0%) (Fig. 8).

#### 4.5.12 Depression

Depression was noted in 4 studies and showed no significant effect overall (SMD: −0.30, Cl: [−0.77, 0.18], p = 0.22, I^2^ = 84%). Leave one out analysis excluding Widmer 2017 reduced heterogeneity (SMD: −0.06, Cl: [−0.41, 0.29], p = 0.75, I^2^ = 65%) (Fig. 8).

Subgroup analysis by intervention type showed a significant difference between active comparator/CBCR and passive usual care for depression at 3 months (p < 0.0001). Active comparator/CBCR interventions (2 studies, N = 121) showed a significant reduction in depression (SMD = −0.81, 95% CI −1.19 to −0.44; I^2^ = 0%), while passive usual care (2 studies, N = 440) showed no significant effect (SMD = 0.08, 95% CI −0.10 to 0.27; I^2^ = 0%). Overall, there was no significant effect on depression (SMD = −0.30, 95% CI −0.77 to 0.18; p = 0.22), with substantial heterogeneity (I^2^ = 84%) (Supplementary Fig. 8).

## 5 Discussion

Cardiovascular diseases are still the major cause of death in the world [15]. Thus, long-term secondary prevention strategies after PCI are now a priority. In the last decade, the HBCTR model has become an innovative approach that aims to improve the accessibility, participation, and adherence to traditional cardiac rehabilitation (CR) [16,17]. CR has been suggested for secondary prevention after PCI by the American Heart Association and American College of Cardiology [18]. The 12 studies (1392 patients) included in this meta-analysis provided the most recent evaluation of HBCTR and its importance in CR.

Recent evidence endorses cardiac rehabilitation for all eligible patients following PCI [[18], [19], [20]]. These findings recognize home- and technology-assisted programs as acceptable alternatives for patients unable to attend CR. They highlight digital platforms, wearable devices, remote monitoring systems, and virtual coaching as emerging tools that support lifestyle modification. In alignment with these evidence, our meta-analysis demonstrates that HBCTR is associated with better improvements in functional capacity (6MWT, VO_2_ max), but modest reductions in systolic blood pressure, better medication adherence, and potential enhancement in LVEF, although this finding was based on limited evidence from two studies. QoL domains showed variable improvements mainly in the short term. The GRADE assessment showed low-to-moderate certainty evidence. However, no consistent benefit is seen for mental health outcomes, lipids, or diastolic blood pressure. Subgroup analyses revealed no overall differences in effect size, and effects were generally similar across subgroups. HBCTR is a promising add-on to routine care after PCI for functional outcomes and adherence outcomes, but evidence is limited by heterogeneity and low certainty due to study variability, and larger trials are needed.

Earlier investigations reported that walking is the best way for stage II rehabilitation of patients with AMI [21,22]. Our study showed an improvement in 6MWT. Notably, HBCTR produced a moderate enhancement of approximately 27 m over standard care compared to some previous reports [23,24]. This indicates that while HBCTR is effective, the magnitude of functional gain may be influenced by the less-structured nature of home environments compared to supervised center-based programs. The improvement in 6MWT is expected, as most of HBCTR models included in our analysis emphasized home-based walking and continuous monitoring via mobile applications. The observed heterogeneity across studies could be justified by the diverse technological delivery models used, ranging from simple SMS-based messaging to complex wearable-integrated platforms. Furthermore, variability in exercise prescription intensity and the level of remote professional supervision across the included studies may account for the mixed results seen in diastolic blood pressure and QoL domains. However, our finding aligned with prior evidence, which revealed that patients in the HBCTR group demonstrated significant improvements in exercise endurance compared with those receiving usual care. Besides, Chen et al. [25] and Ding et al., [26] who suggested 6MWT was a simple, tolerable, and alternative approach to evaluate functional capabilities [24]. Importantly, HBCTR was accompanied with improved exercise capacity and cardiorespiratory fitness primarily through exercise prescription. A previous meta-analysis revealed that the HBCTR is one of the promisingly effective cardiac rehabilitation strategies that improve cardiorespiratory fitness and reduce cardiovascular disease risk factors [27].

Exercise capacity is a vital measure of the effectiveness of CR in patients with coronary heart disease [27]. Our study showed significant improvement in VO_2_ max. Similarly, another study showed that the HBCTR sustain the gains in VO_2_ peak compared to the usual care group [28]. However, variability in equipment availability and exercise prescription across different HBCTR models contributed to the observed heterogeneity in our study, but the overall positive effect provides potential credential to the efficacy of HBCTR.

Our analysis also revealed a potential improvement in LVEF among participants receiving HBCTR. However, this result should be interpreted with caution given the historical inconsistent findings regarding CR effects in short-term cardiac function. In contrast, the improvements in functional capacity (VO_2_ max and 6MWT) provide more potential evidence for the efficacy of HBCTR in the immediate post-PCI phase.

A previous investigation reported that exercise training had no effect on short-term cardiac function improvement [29]. In contrast, other studies revealed that exercise could increase coronary blood flow and myocardial oxygen supply, resulting in improved coronary flow reserve capacity [30,31]. Thus, the consistency observed in our pooled analysis may reflect the structured self-management emphasized in modern HBCTR programs. HBCTR produced modest but potential reductions in systolic blood pressure (BP). This is aligned with prior evidence [32,33]. Our finding is due to improved adherence and lifestyle changes, while diastolic BP findings were inconsistent across primary and sensitivity analyses, limiting confidence in a definitive effect. However, leave-one-out analysis produced significant pooled findings after excluding Widmer 2017. This effect may influenced by baseline therapy and program intensity. Lipid outcomes were unchanged in the pooled analyses, which is consistent with a previous meta-analysis [34]. However, longer-term results from single studies at nine months showed potential favorable effects on LDL-C and total cholesterol, suggesting that any lipid-modifying effects of HBCTR could be time-dependent and warrant further evaluation in larger trials with longer follow-up periods. This finding reflects the limited impact of exercise-based CR on lipid levels compared with pharmacologic therapy, with variability across studies due to differences in baseline lipids, medication regimens, and follow-up duration.

An Improvement in QoL remains an important therapeutic goal in CHD management, although the mechanism of this prognostic effect is not completely understood. Our study showed that QoL outcomes were mixed and not consistent. Physical component scores had significant improvement at three months, and there was limited evidence at nine months, indicating potential for reduction of benefit over time. A meta-analysis of CR programs found a significant benefit on the physical component at six months compared with usual care [35]. Notably, mental component scores showed borderline short-term improvement, although findings were inconsistent and highly heterogeneous. This may reflect the limited psychological content across many HBCTR models, which focused mainly on physical activity and health education rather than psychotherapy. However, psychological improvement often occurs naturally, with strong benefits in remote CR when counseling modules are included [36,37]. Importantly, our study showed that HBCTR improves medication adherence through digital engagement, but anxiety and depression remain unchanged, highlighting the need for stronger mental-health components.

Telemedicine and mHealth systems are now more deeply embedded in cardiovascular care and have an important place in primary and secondary prevention initiatives over the last decade. Digital health interventions, such as mobile apps, wearable devices, and remote monitoring systems have shown to help improve medication adherence, promote lifestyle changes, boost physical activity, and facilitate long-term cardiovascular risk reduction. More recent evidence [38] has emphasized the wide-ranging implications and future potential of these technologies in the context of healthcare delivery [5]. Furthermore, a prior meta-analysis of the available evidence in the field of patient management following acute coronary syndrome (ACS) found that digital health interventions after ACS could positively influence patient engagement, optimize secondary prevention, and decrease the risk of rehospitalization and adverse cardiovascular outcomes after PCI and ACS.

The LIGHT randomized clinical trial series has also provided further evidence of the potential of mHealth-based interventions for cardiovascular prevention. A sub-study of the LIGHT trial showed the positive impact of interventions with smart devices on exercise capacity and functional performance [6]. Likewise, Hayıroğlu et al. pointed out the possible influence of these technologies on autonomic and cardiovascular regulation [39]. Furthermore, another sub-study of LIGHT trial demonstrated that greater digitally monitored physical activity levels were predictive of better estimated atherosclerotic cardiovascular disease risk over time [40]. These results confirm the increasing importance of digital health technologies as part of a broader cardiovascular prevention and rehabilitation program.

### 5.1 Strengths and limitations

This study has several strengths. It is one of the most comprehensive meta-analyses focusing on HBCTR after PCI. Besides, it included diverse demographics and multiple clinically beneficial outcomes. Advanced methodological approaches were followed, including comprehensive database searches, risk-of-bias assessment, subgroup analyses, and leave-one-out sensitivity analyses to address heterogeneity in our study. Besides, GRADE assessment was incorporated to evaluate the certainty of our evidence. Moreover, the inclusion of both physiological and behavioral outcomes provides an in-depth evaluation on the efficacy of HBCTR for secondary prevention after PCI. However, some limitations should be acknowledged. First, heterogeneity was observed in several outcomes, which is due to variations in intervention design, follow-up periods, and technology platforms. The intensity of HBCTR varied from simple messaging apps to wearable-integrated platforms, which resulted in several challenges regarding direct comparisons. Some outcomes were reported by a small number of studies, which could limit the generalizability of these outcomes. Psychological outcomes and lipid parameters have short follow-up durations, which restrict the ability to detect long-term impacts. Furthermore, some studies lacked detailed protocols, and one study had a high risk of bias, which may affect pooled estimates. The GRADE evaluation demonstrated low-to-moderate evidence. Therefore, these results should be carefully interpreted and well-designed; RCTs are required to validate the effects seen in this study.

## 6 Clinical implications and future directions

The clinical implications of our findings are crucial. HBCTR appears to be a promising and potentially effective alternative for selected patients unable to access center-based rehabilitation. However, improvements in exercise capacity, blood pressure, LVEF, and medication adherence support the integration of HBCTR into routine care after PCI. The absence of lipid and psychological advantages suggest that HBCTR should be incorporated with individualized medical therapy and dedicated mental health modules to achieve secondary prevention. Upcoming investigations should focus on standardizing HBCTR protocols. In addition, we recommend to integrate more behavioral and psychological components, and evaluate long-term cardiovascular outcomes, such as recurrent adverse events and hospitalization.

## 7 Conclusion

Our study showed that HBCTR improves functional capacity, cardiopulmonary fitness, systolic blood pressure, left ventricular ejection fraction, and medication adherence after percutaneous coronary intervention. However, the effects on lipids and psychological outcomes are variable. Importantly, home based rehabilitation showed a promising alternative for secondary prevention after coronary intervention, particularly for improving functional capacity and medication adherence. However, its impact on lipid parameters and psychological well-being remains inconclusive, due to a lack of dedicated counseling modules in current models. Therefore, upcoming standardized protocols are required to confirm long-term cardiovascular advantages.

## Data availability statement

Data sharing is not applicable to this article as no new data were created or analyzed beyond publicly available published studies included in this systematic review and meta-analysis.

## Funding

None.

## CRediT authorship contribution statement

**Sanjeet Kumar:** Conceptualization, Data curation, Project administration, Writing – original draft, Writing – review & editing. **Fnu Venjhraj:** Data curation, Writing – original draft, Writing – review & editing. **Ahmed W. Hageen:** Formal analysis, Methodology, Writing – original draft, Writing – review & editing. **Javeria Farooq:** Data curation, Validation, Visualization, Writing – review & editing. **Meva Ram:** Data curation, Writing – review & editing. **Fnu Sahil:** Data curation, Writing – original draft, Writing – review & editing. **Sandeep Kumar:** Investigation, Validation, Writing – review & editing. **Jugdesh Kumar:** Methodology, Software, Writing – original draft, Writing – review & editing. **Muhammad Umar:** Formal analysis, Methodology, Writing – review & editing. **Ravi Das:** Visualization, Writing – review & editing. **Sahil Jairamani:** Visualization, Writing – review & editing. **Mirza Mohammad Ali Baig:** Writing – review & editing. **Carol Wright Becker:** Supervision.

## Declaration of interests

All authors are requested to disclose any actual or potential conflict of interest including any financial, personal or other relationships with other people or organizations within three years of beginning the submitted work that could inappropriately influence, or be perceived to influence, their work. If there are no conflicts of interest, the COI should read: “The authors report no relationships that could be construed as a conflict of interest”.

Signature of all the Authors:

S. Kumar, F. Venjhraj, A.W. Hageen, J. Farooq, M. Ram, F. Sahil.

S. Kumar.

J. Kumar, M. Umar, R. Das, S. Jairamani, M.M.A. Baig.

C.W.Becker.
