# Supplementary material for: Effectiveness of home-based cardiac telerehabilitation programs in improving clinical outcomes after PCI: A systematic review and meta-analysis of randomized and non-randomized studies
Source: Int J Cardiol Cardiovasc Risk Prev. 2026 Jul 7;30:200679. doi: 10.1016/j.ijcrp.2026.200679 (PMC13380744; doi:10.1016/j.ijcrp.2026.200679)
Supplement: Multimedia component 1 [file mmc1.docx]

**Supplementary material**

**Title:**

**Effectiveness of Home-Based Cardiac Telerehabilitation Programs in Improving Clinical Outcomes after PCI: A Systematic Review and Meta-Analysis of Randomized and Non-Randomized Studies**

**Index:**

1. Supplementary Appendix 1.1: Detailed Search Strategy
    1.1.1 Databases Searched
    1.1.2 PubMed Search Strategy
    1.1.3 Google Scholar Search Strategy
    1.1.4 Additional Search Methods
2. Supplementary Appendix 1.2: PRISMA 2020 Flowchart
3. Supplementary Table 1: Study Characteristics and Participants' characteristics
4. Supplementary Table 2: GRADE assessment
5. Supplementary Figure 7: Forest plot for subgroup analysis of physical component summary (PCS) quality of life at 3 months by intervention type.
6. Supplementary Figure 9: Forest plot for subgroup analysis of mental component summary (MCS) quality of life at 3 months by intervention type.
7. Supplementary Figure 11: Forest plot for subgroup analysis of systolic blood pressure at 3 months by intervention type.
8. Supplementary Figure 14: Forest plot for subgroup analysis of total cholesterol at 3 months by intervention type.
9. Supplementary Figure 16: Forest plot for subgroup analysis of triglycerides (TG) at 3 months by intervention type.
10. Supplementary Figure 18: Forest plot for subgroup analysis of low-density lipoprotein cholesterol (LDL-C) at 3 months by intervention type.
11. Supplementary Figure 20: Forest plot for subgroup analysis of high-density lipoprotein cholesterol (HDL-C) at 3 months by intervention type.
12. Supplementary Figure 24: Forest plot for subgroup analysis of depression at 3 months by comparator type.

**Supplementary Appendix 1.1: Detailed Search Strategy**

**1.1.1 Databases Searched** The following databases were systematically searched: PubMed, Google Scholar, and the Cochrane Library to September 1, 2025.

**1.1.2 PubMed Search Strategy** (("Percutaneous Coronary Intervention"[Mesh] OR "Coronary Angioplasty"[Mesh] OR PCI OR "percutaneous coronary intervention" OR "coronary angioplasty" OR "myocardial infarction")

 AND

("Telerehabilitation"[Mesh] OR "Cardiac Rehabilitation"[Mesh] OR "home-based cardiac rehabilitation" OR "home cardiac rehabilitation" OR "cardiac telerehabilitation" OR HBCTR OR telemedicine OR mHealth OR eHealth OR wearable OR smartphone OR WeChat))
 AND
 (randomized controlled trial OR controlled clinical trial OR RCT OR CCT)

 **1.1.3 Google Scholar Search Strategy** ("percutaneous coronary intervention" OR PCI OR "coronary angioplasty" OR "myocardial infarction")

AND

("home-based cardiac rehabilitation" OR "home cardiac rehabilitation" OR "cardiac telerehabilitation" OR HBCTR OR telerehabilitation OR telemedicine OR mHealth OR eHealth OR wearable OR smartphone OR WeChat)

AND

("randomized controlled trial" OR RCT "controlled clinical trial" OR CCT)

First 300 results screened (sorted by relevance)

**1.1.4 Additional Search Methods** Manual screening of reference lists of included studies. Screening of related articles

**Supplementary Appendix 1.2: PRISMA 2020 Flowchart**



**Identification of studies via databases and registers**

**Identification**

Records identified through
Databases searching (n=2888)

Pubmed(n=1373)

Google Scholar(n=1187)

Cochrane(n=328)

Records removed before screening:
Duplicate records removed

(n=1143)
Records marked as ineligible by automation tools (n =341)
Records removed for other reasons (n= 2)

**Screening**

Records screened (n=1402)

Records excluded (n =1376)

Reports assessed for eligibility (n=26)

Full-text articles excluded based on (n=14)
Protocol only (n=4)
Irrelevant outcomes(n=5)

Cross over trial(n=2)

Different follow ups(n=3)

**Included**

Studies included in quantitative synthesis (meta-analysis) (n =12)

**Supplementary Table 1: Study Characteristics and Participants' characteristics**

| **Author, Year, Country** | **Design** | **Total (n)** | **IG (n, Male %)** | **CG (n, Male %)** | **Age IG (Mean ± SD)** | **Age CG (Mean ± SD)** | **Intervention** | **Control** | **Duration** | **Outcomes** |
| --- | --- | --- | --- | --- | --- | --- | --- | --- | --- | --- |
| Widmer et al., 2017 (USA) | RCT | 71 | 29 (78%) | 29 (85%) | 62.5 ± 10.7 | 63.6 ± 10.9 | DHI + CR | Standard CR | 3 months | BP (SBP and DBP), lipid profile, Vo2max, QoL (overall), Depression |
| Dorje et al., 2019 (China) | RCT | 312 | 156 (70%) | 156 (70%) | 59.1 ± 9.4 | 61.9 ± 8.7 | SMART-CR/SP + WeChat | Usual care | 12 months | 6MWT, BP (SBP), lipid profile, QoL (PCS and MCS), Medication adherence, Depression, Anxiety. |
| Lee et al., 2013 (Korea) | RCT | 55 | 26 (85%) | 29 (76%) | 54.3 ± 8.9 | 57.3 ± 7.5 | HBCTR with pedometer + counselling | Usual care | 3 months | BP (SBP and DBP), Quality of life, exercise capacity |
| Fang, 2019 (China) | RCT | 67 | 33 (63.6%) | 34 (61.8%) | 60.2 ± 9.3 | 61.4 ± 10.7 | HBCTR via belt strap, smartphone, and web port | Usual care | 1.5 months | 6MWT, BP (systolic blood pressure, diastolic blood pressure); anxiety and depression (CDS score); risk factors (FTND score); quality of life (SF-36 (PCS), SF-36 (MCS). |
| Li, 2022 (a) (China) | RCT | 80 | 40 (67.5%) | 40 (62.5%) | 55.4 ± 8.9 | 55.6 ± 8.3 | HBCTR via WeChat group and exercise bracelet | Outpatient rehabilitation | 6 months | 6MWT, QoL (overall), Medication adherence. |
| Zheng et al., 2024 (China) | RCT | 106 | 53 (60%) | 53 (56.6%) | 63.5 ± 9.5 | 63.0 ± 10.4 | HBCTR via wearing an intelligent sports bracelet. | Outpatient care and telephone follow-up | 3 months | 6MWT, quality of life (SF-36 (PCS), SF-36 (MCS), Vo2max, LVEF, anaerobic threshold. |
| Cruz-Cobo et al., 2024 (Spain) | RCT | 300 | 150 (68.7%) | 150 (69.3%) | 61.1 ± 8.7 | 63.9 ± 8.4 | HBCTR via eMOTIVA app installed on their mobile phones or tablets. | Usual care | 3 months | BP (SBP and DBP), lipid profile, adherence to the Mediterranean diet, exercise capacity measured with the 6MWT, Cardiovascular risk factors. |
| Bernal-Jiménez et al., 2021 (Spain) | RCT | 128 | 67 (79%) | 61 (64%) | 57.7 ± 8.2 | 61.5 ± 9.5 | mHealth group with app (EVITE project) in their phones which monitor food consumption, blood pressure, smoking, and therapeutic adherence. | Standard care | 9 months | BP (systolic and diastolic); anxiety and depression (CDS score); risk factors (FTND score); quality of life (SF-36 (PCS), SF-36 (MCS); diet, frequency of food intake, Physical activity performed, smoking, knowledge of a healthy lifestyle |
| Xiaojie Li et al., 2023 (China) | RCT | 50 | 26 (46.2%) | 24 (50%) | 66.0 ± 3.8 | 66.6 ± 4.4 | HBCTR via wearable smart device with 5G IOT CR intelligence platform and Healthy Life Cycle application (app) | Traditional in-hospital CR training. | 3 months | Lipid profile, Vo2max, anxiety and depression. |
| Li et al., 2022(b) (China) | RCT | 95 | 47 (74.4%) | 48 (81.3%) | 65.3 ± 8.7 | 67.7 ± 7.6 | Home-based online supervised exercise program (HOSEP) and WeChat group | Conventional health education and home exercise program booklet | 1.5 months | 6 MWT, Lipid profile, BP (systolic and diastolic). |
| Dehghani et al., 2024 (Iran) | RCT | 80 | 40 (47.5%) | 40 (50%) | 49.8 ± 7.9 | 51.5 ± 7.5 | Home-based CR (telerehabilitation) | Traditional center-based CR | 2 months | health-related quality-of-life (PCS and MCS) BP (systolic and diastolic), LVEF. |
| Lee et al., 2017 (Korea) | CCT | 48 | 22 (68.2%) | 26 (84.6%) | 55.9 ± 6.2 | 56.1 ± 7.0 | Smart phone with "Our Family Heart Health Guardian" app, Smart band, KakaoTalk messages | Center-based CR | 3 months | quality of life (SF-36 (PCS), SF-36 (MCS); medication adherence, regular exercise, stress, calorie intake, lipids, cholestero, |

*G=Intervention group; CG=control group; DHI=Digital health interventions; CR=cardiac rehabilitation; LVEF= Left ventricular ejection fraction; QoL=quality of life; SMART-CR/SP=a system involved smartphone-based home cardiac rehabilitation and secondary prevention program; 6MWT = six-minute walking test; HBCTR home-based cardiac telerehabilitation; SF-36 (MCS) SF-36 Health Survey (mental component summary scale); SF-36 (PCS) SF-36 Health Survey (physical component summary scale); IOT=internet of things; RCT= Randomized controlled trial; CCT=Controlled clinical trial.*

**Supplementary Table 2: GRADE assessment**

| **Certainty assessment** | | | | | | | **№ of patients** | | **Effect** | | **Certainty** | **Importance** |
| --- | --- | --- | --- | --- | --- | --- | --- | --- | --- | --- | --- | --- |
| **№ of studies** | **Study design** | **Risk of bias** | **Inconsistency** | **Indirectness** | **Imprecision** | **Other considerations** | **HBCTR** | **Usual care** | **Relative (95% CI)** | **Absolute (95% CI)** |  |  |
| **6-minute walking test (6MWT)** | | | | | | | | | | | | |
| 5 | randomized trials | serious | serious | not serious | not serious | none | 329 | 331 | - | MD **27.38 higher** (11.28% higher to 43.49% higher) | ⨁⨁◯◯ Low-B |  |

| **QOL-PCS** | | | | | | | | | | | | |
| --- | --- | --- | --- | --- | --- | --- | --- | --- | --- | --- | --- | --- |
| 5 | randomized trials | very serious | serious | not serious | serious | none | 304 | 309 | - | SMD **0.38 higher** (0.06 higher to 0.71 higher) | ⨁◯◯◯ Very low a, b, and c. |  |
| **QOL-MCS** | | | | | | | | | | | | |
| 5 | randomized trials | very serious | very serious | not serious | serious | none | 304 | 309 | - | SMD **0.35 higher** (0.01 higher to 0.7 higher) | ⨁◯◯◯ Very low: a, d, e. |  |

| **SBP at 3 months** | | | | | | | | | | | | |
| --- | --- | --- | --- | --- | --- | --- | --- | --- | --- | --- | --- | --- |
| 7 | randomized trials | serious | not serious^b^ | not serious | not serious | none | 489 | 491 | - | MD **3.01 lower** (5.75 lower to 0.26 lower) | ⨁⨁⨁◯ Moderate^a,b^ |  |

| **Diastolic BP** | | | | | | | | | | | | |
| --- | --- | --- | --- | --- | --- | --- | --- | --- | --- | --- | --- | --- |
| 6 | randomized trials | serious | very serious^b^ | not serious | serious | none | 333 | 335 | - | MD **0.1 lower** (3.31 lower to 3.12 higher) | ⨁◯◯◯ Very low a, b, and c. |  |

**CI:** confidence interval; **MD:** mean difference

| **LVEF** | | | | | | | | | | | | |
| --- | --- | --- | --- | --- | --- | --- | --- | --- | --- | --- | --- | --- |
| 2 | randomized trials | serious | not serious | not serious | not serious | none | 93 | 93 | - | MD **2.03 higher** (0.15 higher to 3.91 higher) | ⨁⨁⨁◯ Moderate |  |

| **VO₂ MAX** | | | | | | | | | | | | |
| --- | --- | --- | --- | --- | --- | --- | --- | --- | --- | --- | --- | --- |
| 3 | randomized trials | serious | very serious^b^ | not serious | serious | none | 116 | 111 | - | MD **2.55 higher** (0.42 higher to 4.68 higher) | ⨁◯◯◯ Very low a, b, and c. |  |

| **Total cholesterol at 3 months** | | | | | | | | | | | | |
| --- | --- | --- | --- | --- | --- | --- | --- | --- | --- | --- | --- | --- |
| 5 | randomized trials | serious | serious^b^ | not serious | not serious | none | 416 | 412 | - | MD **0.06 higher** (0.2 lower to 0.32 higher) | ⨁⨁◯◯ Low-B |  |

| **TG at 3-month follow-up** | | | | | | | | | | | | |
| --- | --- | --- | --- | --- | --- | --- | --- | --- | --- | --- | --- | --- |
| 5 | randomized trials | serious | serious^b^ | not serious | serious | none | 416 | 412 | - | MD **0.11 lower** (0.34 lower to 0.11 higher) | ⨁◯◯◯ Very low a, b, and c. |  |

| **Depression** | | | | | | | | | | | | |
| --- | --- | --- | --- | --- | --- | --- | --- | --- | --- | --- | --- | --- |
| 4 | randomized trials | serious | serious^b^ | not serious | not serious | none | 286 | 275 | - | SMD **0.3 lower** (0.77 lower to 0.18 higher) | ⨁⨁◯◯ Low-B |  |

| **LDL-C at 3 months** | | | | | | | | | | | | |
| --- | --- | --- | --- | --- | --- | --- | --- | --- | --- | --- | --- | --- |
| 5 | randomized trials | serious | serious^b^ | not serious | not serious | none | 416 | 412 | - | MD is **0.12 higher** (0.17 lower to 0.41 higher) | ⨁⨁◯◯ Low-B |  |

| **Medication adherence** | | | | | | | | | | | | |
| --- | --- | --- | --- | --- | --- | --- | --- | --- | --- | --- | --- | --- |
| 3 | randomized trials | serious | serious^b^ | not serious | not serious | none | 158/238 (66.4%) | 121/235 (51.5%) | **OR 1.98** (1.23 to 3.18) | **163 more per 1,000** (from 51 more to 257 more) | ⨁⨁◯◯ Low-B |  |

| **Anxiety** | | | | | | | | | | | | |
| --- | --- | --- | --- | --- | --- | --- | --- | --- | --- | --- | --- | --- |
| 3 | randomized trials | serious | very serious^b^ | not serious | serious | none | 249 | 241 | - | SMD **0.22 lower** (0.74 lower to 0.3 higher) | ⨁◯◯◯ Very low a, b, and c. |  |

| **HDL-C** | | | | | | | | | | | | |
| --- | --- | --- | --- | --- | --- | --- | --- | --- | --- | --- | --- | --- |
| 5 | randomized trials | serious | not serious | not serious | serious^b^ | none | 416 | 412 | - | MD **0.01 lower** (0.05 lower to 0.03 higher) | ⨁⨁◯◯ Low-B |  |

**CI:** confidence interval; **MD:** mean difference

**Supplementary Figure 1: Forest plot for subgroup analysis of physical component summary (PCS) quality of life at 3 months by intervention type.**

**
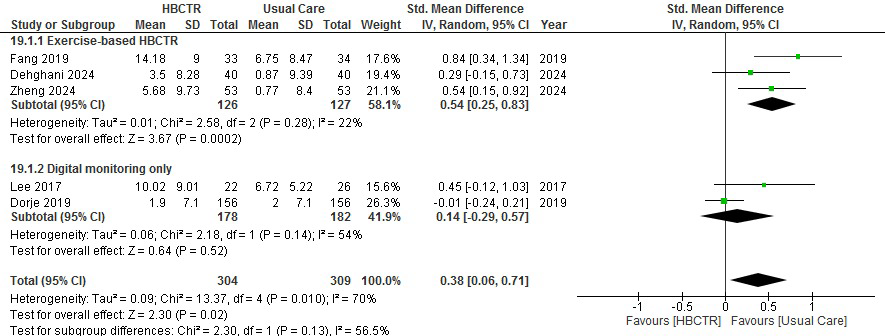
**

**Supplementary Figure 2: Forest plot for subgroup analysis of mental component summary (MCS) quality of life at 3 months by intervention type.**

**
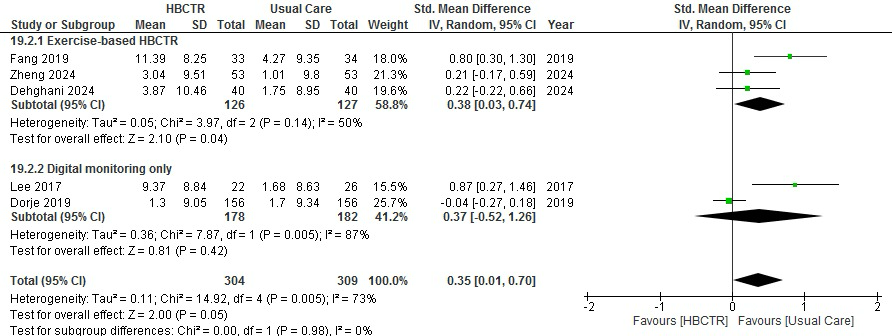
**

**Supplementary Figure 3: Forest plot for subgroup analysis of systolic blood pressure at 3 months by intervention type**.

**
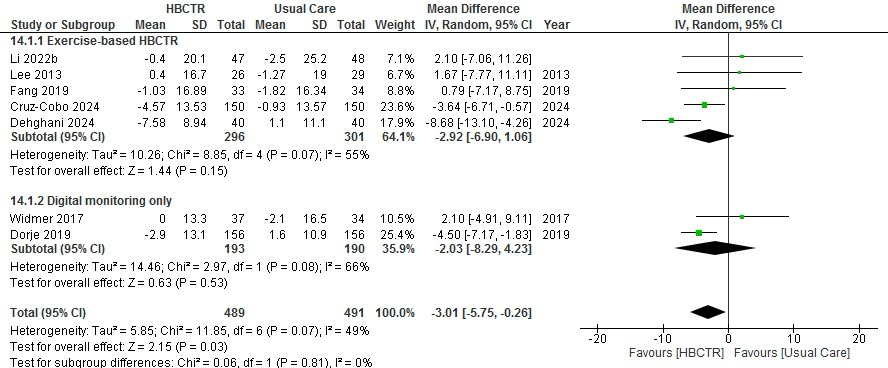
**

**Supplementary Figure 4: Forest plot for subgroup analysis of total cholesterol at 3 months by intervention type.**

**
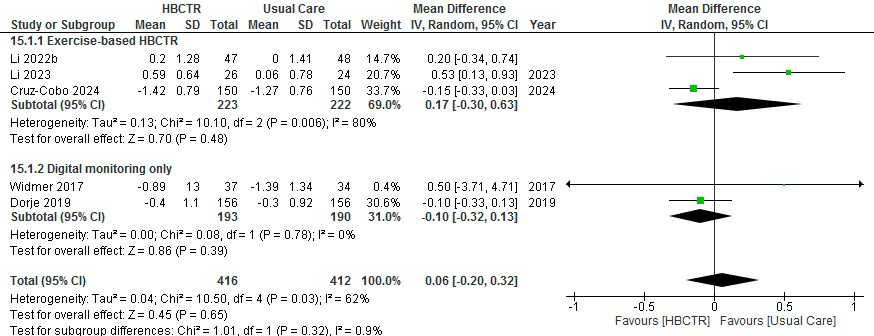
**

**Supplementary Figure 5: Forest plot for subgroup analysis of triglycerides (TG) at 3 months by intervention type.**

**
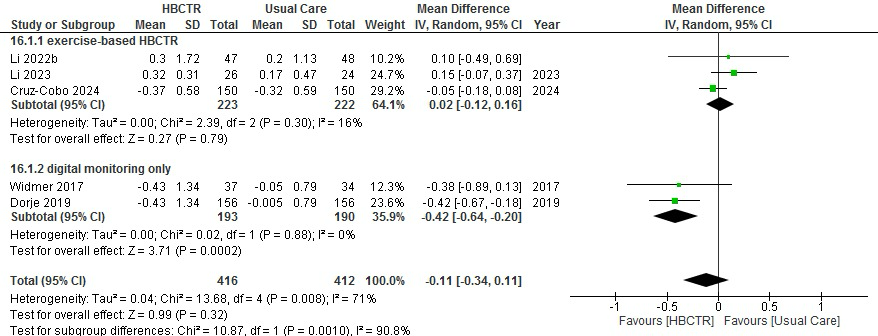
**

**Supplementary Figure 6: Forest plot for subgroup analysis of low-density lipoprotein cholesterol (LDL-C) at 3 months by intervention type.**

**
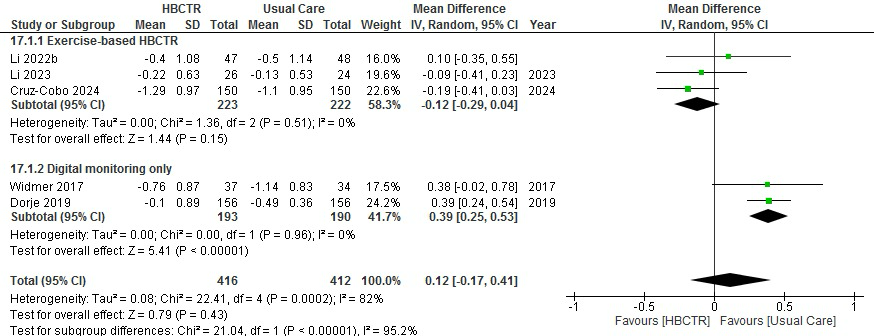
**

**Supplementary Figure 7: Forest plot for subgroup analysis of high-density lipoprotein cholesterol (HDL-C) at 3 months by intervention type.**

**
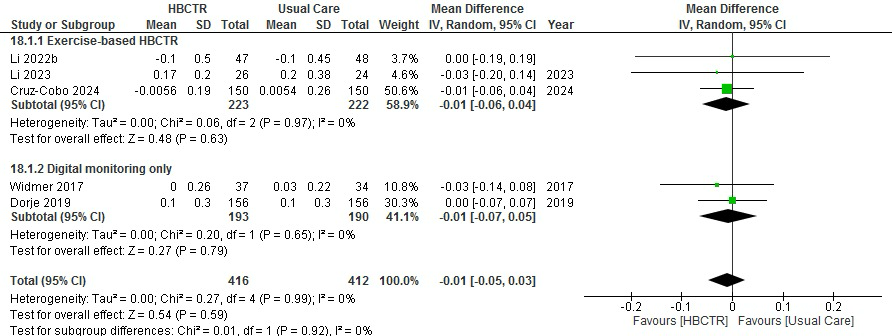
**

**Supplementary Figure 8: Forest plot for subgroup analysis of depression at 3 months by comparator type.**

**
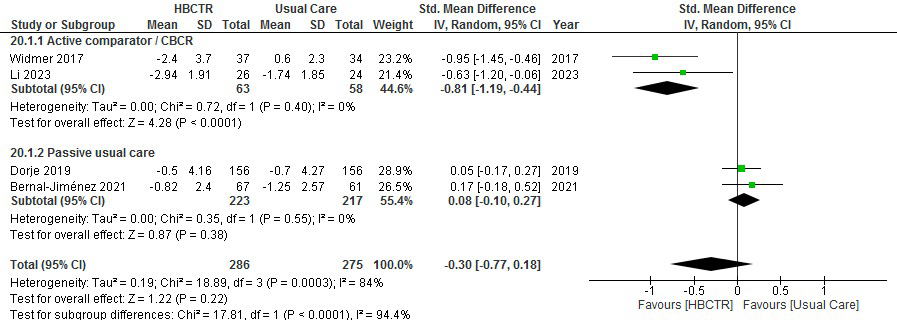
**
